# Mucin 1 promotes tumor progression through activating WNT/β-catenin signaling pathway in intrahepatic cholangiocarcinoma

**DOI:** 10.7150/jca.63235

**Published:** 2021-10-03

**Authors:** Fei Song, Fei-Yu Chen, Sui-Yi Wu, Bo Hu, Xiao-liang Liang, Hao-Qin Yang, Jian-Wen Cheng, Peng-Xiang Wang, Wei Guo, Jian Zhou, Jia Fan, Zhong Chen, Xin-Rong Yang

**Affiliations:** 1 Department of Hepatobiliary Surgery, Affiliated Hospital of Nantong University, Nantong 226001, P. R. China;; 2 Department of Liver Surgery & Transplantation, Liver Cancer Institute, Zhongshan Hospital, Fudan University, Key Laboratory of Carcinogenesis and Cancer Invasion, Ministry of Education, Shanghai 200032, P. R. China;; 3 Transfusion Department, Zhongshan Hospital, Fudan University, Shanghai 200032, P. R. China;; 4 Nanjing Foreign Language School, Nanjing 210018, P. R. China;; 5 Department of Laboratory Medicine, Zhongshan Hospital, Fudan University, Shanghai 200032, P. R. China;; 6 Institutes of Biomedical Sciences, Fudan University, Shanghai 200032, P. R. China

**Keywords:** intrahepatic cholangiocarcinoma, mucin 1, Wnt/β-catenin pathway

## Abstract

**Background:** Current treatment options for intrahepatic cholangiocarcinoma (ICC) are limited by the lack of understanding of the disease pathogenesis. It has been known that mucin 1 (MUC1) is a cell surface mucin that highly expressed in various cancer tissues. However, its role in ICC has not been well studied. The purpose of this study was to investigate the clinical significance and biological function of MUC1 in ICC.

**Methods:** qRT-PCR and western blot assays were performed to examine MUC1 expression. RNA-Seq (RNA Sequencing) s conducted to explore the RNA expression. A tissue microarray study including 214 ICC cases was also conducted to evaluate the clinical relevance and prognostic significance of MUC1. The role and underlying mechanisms of MUC1 in regulating cell growth and invasion were further explored both *in vitro* and *in vivo* models.

**Results:** The mRNA and protein levels of MUC1 were significantly up-regulated in ICC compared to paired non-tumor tissues. Depletion of MUC1 in HCCC9810 cells significantly inhibited cell proliferation, migration and invasion *in vitro* and overexpression of MUC1 in RBE cells resulted in increased cell proliferation, migration and invasion. Both univariate and multivariate analysis revealed that the protein expression of MUC1 was associated with overall survival and relapse-free survival after tumor resection. Clinically, high MUC1 expression was more commonly observed in aggressive tumors. Further studies indicated that MUC1 exerted its function through activating Wnt/ β-catenin pathway.

**Conclusions:** Our data suggests that MUC1 promoted ICC progression via activating Wnt / β-catenin pathway. This study not only deciphered the role of MUC in ICC pathogenesis, but also shed light upon identifying novel potential therapeutic targets.

## Introduction

Intrahepatic cholangiocarcinoma (ICC) is the second most common primary liver malignancy and often progresses aggressively 1,2. The morbidity and mortality of CC have increased rapidly in recent years 3,4. Although surgical resection is still the first-line treatment, most patients may already miss the optimal surgical window and even metastasized upon the diagnosis. Currently, no effective chemotherapy or targeted molecular therapy has been identified for ICC, mainly due to the insufficient understanding of its pathogenesis.

Mucin (MUC) is the main component in mucus secretion and has unique biophysical and chemical properties due to its nature and glycosylation condition 5,6. It has been known that MUC can participate in cell regeneration, differentiation, integration, signaling, adhesion, and apoptosis under different conditions 7,8. Human mucins can be divided into two subgroups based on structure, function, and cell localization 9: membranes (associated with the cell surface) and secreted 10-12. In the mucin family, MUC1, MUC4, and MUC16 belong to the membrane binding/transmembrane subgroup 7. Among them, MUC1 has been reported to play a key role as an oncogene in a variety of solid tumors 13-15. Our previous study showed that the mRNA expression of MUC1 was associated with the prognosis of ICC patients 16. However, the clinical significance of MUC1 expression at protein level in ICC patients is still needed to further exploration, especially with long-term follow-up and a large number of patients. Moreover, the role of MUC1 in ICC progression has not been clearly defined.

In this study, we explored the expression of MUC1 in human ICC cell lines and tumor tissues by quantitative real-time PCR (qRT-PCR), Western blot analysis and RNA-Seq (RNA Sequencing) analysis. To evaluate the clinical relevance and prognostic significance of MUC1 in ICC, we further examined the expression of MUC1 in tissue microarrays (TMAs) with 214 ICC patients. The completion of this study will not only help us to better understand the pathogenesis of ICC, but also provide insights developing novel therapeutic targets.

## Materials and methods

### Patients and specimens

A total of 30 pairs of snap-frozen ICC samples and matched adjacent non-tumor tissues samples were collected from ICC patients during curative resection in the Liver Cancer Institute, Zhong Shan Hospital, Fudan University in 2019. Among those, 10 pairs were randomly selected for RNA-Seq (RNA Sequencing) analysis, and 8 pairs were selected to detect MUC1 protein expression. Another 12 ICC samples with or without lymph node metastasis for further validation were also randomly selected from the same tissue bank.

Tumor specimens used in tissue microarrays (TMAs) analysis were obtained from 214 consecutive ICC patients who received liver resection in the Liver Cancer Institute, Zhong Shan Hospital, Fudan University from February 2001 to December 2006. The clinicopathologic characteristics of the 214 ICC patients and 10 mRNA-seq patients were summarized in **Table 1** and **Table S1**, respectively. Inclusion criteria: 1. Postoperative pathological diagnosis was primary intrahepatic cholangiocarcinoma; 2. The patient did not undergo any other treatment before surgery. Exclusion criteria: 1. Postoperative pathological diagnosis of patients with hepatocellular carcinoma, extrahepatic cholangiocarcinoma, or non-primary tumors, such as liver metastases of bowel cancer; 2. Patients undergoing chemotherapy, interventional therapy, targeted therapy or immunization before surgery Treatment and other therapeutic. Histopathological diagnosis was made based on World Health Organization criteria, and tumor staging was defined according to the Seven Edition of Tumor-Node-Metastasis (TNM) Classification of International Union against Cancer 17. Clinical data collection, postoperative follow-up procedures, and admission criteria were performed according to the unified guidelines described in our previous study 18,19. OS was defined as the interval between the date of surgery and the date of death, and RFS was defined as the interval between the date of surgery and the date of tumor recurrence 19. Ethical approval for human subjects was obtained from the research ethics committee of Zhong Shan Hospital, and informed consent was obtained from each patient. The number of Ethical approval in this study is B2018-018(2).

### TMA construction and immunohistochemistry

Tissue microarray blocks were constructed as described previously 20. Briefly, all ICC tissues as well as matched adjacent non-tumor tissues were reviewed by two pathologists and representative tumor areas were pre-marked in the paraffin blocks. Two core biopsies of 1 mm in diameter were taken from the donor blocks and transferred to the recipient paraffin block at defined array positions. Consecutive sections with 4 μm thickness were presented on 3-aminopropyltriethoxysilane-coated slides (Shanghai Biochip). Immunostaining intensities of these markers were semi-quantitatively scored as follows: 0, negative; 1, weak; 2, moderate; 3, strong. The score of immunostaining intensity was assessed by two pathologists independently, and comparisons were performed between tumor/normal samples.

### Cell lines and transfection

This study used two human ICC cell lines, HCCC9810 (Shanghai Branch, Chinese Academy of Sciences, Shanghai, China) and RBE (Cell Resource Center, Tohoku University, Japan). Cells were maintained in RPMI-1640 plus 10% fetal bovine serum (Gibco, Big Island, NY, USA) plus penicillin/streptomycin.

A lentiviral vector encoding wild-type MUC1 was transfected into RBE cells and named RBE-MUC1 cells. RBE-Mock cells transfected with lentiviral vectors only were used as controls. Meanwhile, a lentiviral vector encoding shMUC1 was transfected into HCCC9810 cells and named HCCC9810-shMUC1 cells. HCCC9810-Mock cells transfected with lentiviral vectors only were used as controls. The results of the transfection were verified by qRT-PCR and Western blot analysis.

### Cell proliferation, cell cycle, migration and invasion assays

Cell proliferation, cell cycle, migration and invasion assays were performed as previously described 20. The Cell Counting Kit-8 (Dojindo, Kumamoto, Japan) was used to measure cell proliferation. For the wound-healing assay, a scratch was created across the center of the cell layer using a sterile 100-μl pipette tip. After 48 h, photographs were taken under the microscope, and cell migration was calculated using Image J software. For matrigel invasion assay, 2 × 10^6^ cells from each group were seeded in the upper chamber of the plate and maintained in FBS-free RPMI 1640 and mitomycin C. The chamber was coated with a matrix gel (1:8 diluted, Corning, ME). RPMI 1640 containing 20% FBS was added to the lower chamber as a chemo-attractant. After 48 h of incubation, tumor cells that had invaded to the lower surface of the membrane were fixed using 4% methanol and stained with crystal violet before counting in five random ×100 microscopic fields per sample. Cell cycle was determined by FCM with PI/RNase staining buffer (BD Biosciences). All assays were performed in triplicates and repeated at least twice.

### Western blot, and qRT-PCR assays

Protein was extracted from ICC cells or frozen samples using RIPA buffer and analyzed by Western blot as previously described 21. Total RNA was extracted using Trizol reagent (Invitrogen, Carlsbad, California, USA), and reverse-transcribed into cDNA using PrimeScript RT kit (Takara, Japan). SYBR Premix Ex Taq^TM^ (Takara, Japan) was used for qRT-PCR according to the manufacturer's instructions, and gene amplification and detection were performed using ABI PRISM 7900 sequence detection system (Applied Bio systems, Foster City, CA, USA).

### *In vivo* assays

Male non-obese diabetic severe combined immunodeficiency (NOD/SCID) mice (4 weeks old) were purchased from the Shanghai Institute of Material Medicine of the Chinese Academy of Sciences and raised under conditions free of specific pathogens. HCCC9810-shMUC1, HCCC9810-Mock, RBE-MUC1 and RBE-Mock cells (5 × 10^6^) were suspended in 200 μl of serum-free RPMI-1640 and Matrigel (BD biosciences; 1: 1) and injected subcutaneously into the small Ventral side of the mice. The tumor volume was measured every three days with a caliper and documented in mm^3^. At the end of the experiment, the tumors were collected from the model and measured. Animal care and experimental procedures were performed in accordance with guidelines developed by the Shanghai Medical Laboratory Animal Care Committee. The entire animal experiment was ethically approved by the Research Ethics Committee of Zhongshan Hospital.

### RNA-Seq analysis

The total RNA was extracted using the RNeasy kit (Qiagen, Hilden, Germany) according to the manufacturer's instructions. RNA was quantified using the Qubit® RNA HS Assay Kit by Qubit®2.0 Fluorometer (Life Technologies, Grand Island, NY, USA). RNA quality was determined using an Agilent 2100 bioanalyzer (Agilent Technologies, Palo Alto, CA, USA). RNA-seq library construction for Next-generation sequencing and paired-end deep sequencing was performed on an Illumina PE150 platform (Illumina, San Diego, CA), according to the manufacturer's protocol.

### Statistical analysis

Statistical analysis was performed using SPSS version 23.0 and GraphPad Prism 7.0. The χ^2^ test or Fisher's exact test was used to compare the categorical data, and the Student's t test or one-way analysis of variance was used to analyze the quantitative data. The Kaplan-Meier method was used to plot the OS and RFS curves and compared using a log-rank test. Cox proportional hazard regression models were used for univariate and multivariate analysis. A two-tailed P value of <0.05 was considered statistically significant. The complete dataset is available as NGDC proles on the NGDC (National Genomics Data Center) database (https://ngdc.cncb.ac.cn/search/?dbId=bioproject&q=PRJCA005304).

## Results

### MUC1 was up-regulated in human ICC tissues and associated with lymphatic metastasis

**Figure 1:**
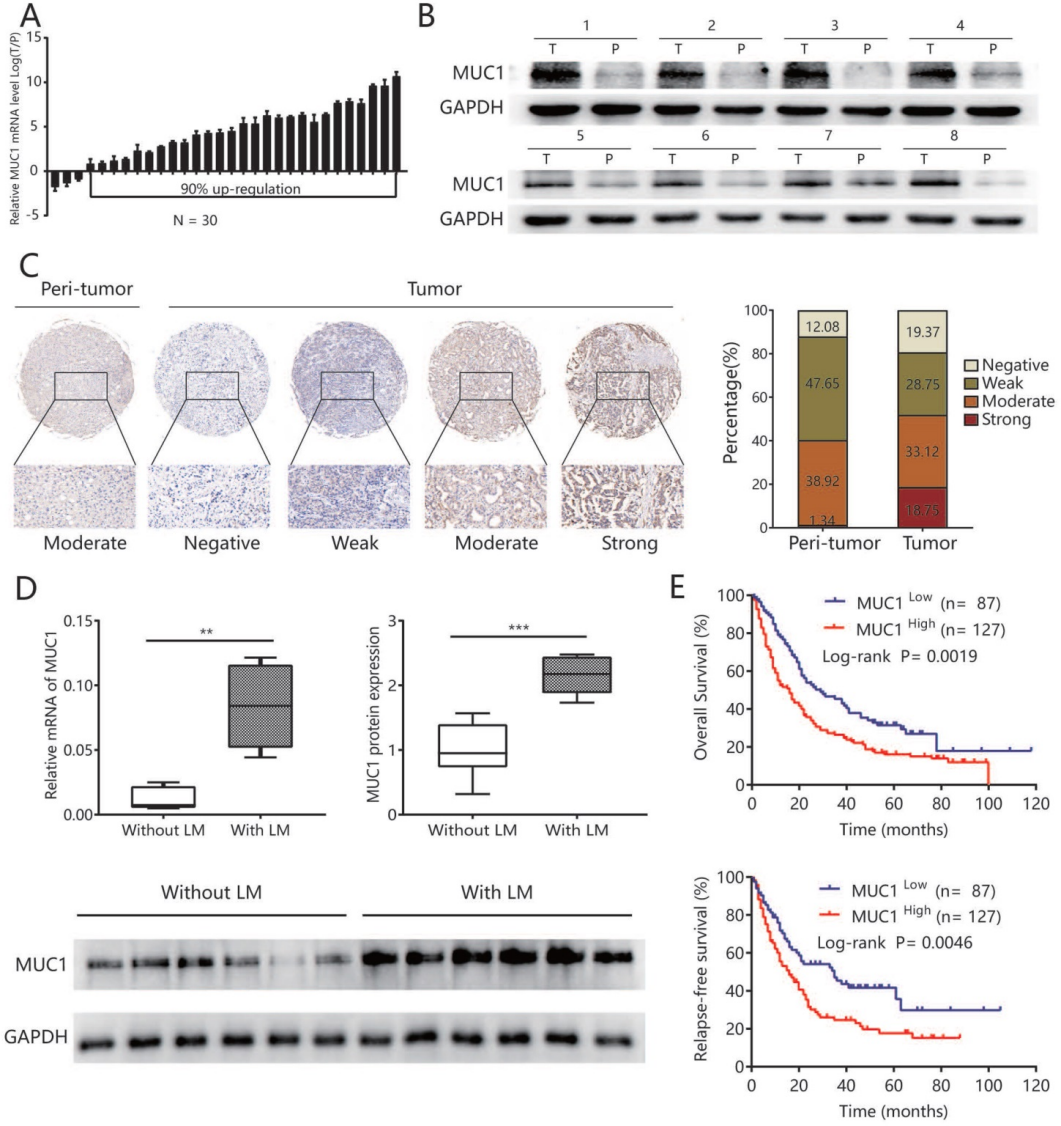
** MUC1 was up-regulated in human ICC tissues and associated with lymphatic metastasis. A.** The mRNA expression of MUC1 in 30 paired ICC tumor and adjacent non-tumor tissues. **B.** The protein expression of MUC1 in 8 paired ICC tumor (T) and adjacent non-tumor tissues (P). **C.** Representative immunostaining images of MUC1 in ICC and adjacent nontumor tissues. Left: adjacent non-tumor tissues. Right: different staining intensities in ICC. Bar graph showed the results for the staining intensity of MUC1 in tissue microarrays containing 214 ICC patients. **D.** The mRNA expression and protein expression of MUC1 in ICC with lymphatic metastasis and without lymphatic metastasis. Densitometry analysis for MUC1 was expressed relative to the loading control, GAPDH. **E.** Kaplan-Meier curves for overall survival and relapse-free survival of ICC patients according to the expression of MUC1. All bar graphs depicted quantification of triplicate results with mean ± SD, **P < 0.01, ***P < 0.001.

To explore the potential role of MUC1 in ICC, we first evaluated the mRNA expression of MUC1 in 30 pairs of ICC samples and matched non-tumor liver tissues. The results showed that in 90% (27/30) of all pairs, the MUC1 mRNA expression was significantly up-regulated in ICC tissues compared to adjacent non-tumor liver tissues (P = 0.039) (**Figure 1A**). Western blot analysis performed on 8 paired ICC tumors and adjacent non-tumor liver tissues showed similar results (**Figure 1B**). Compared with other types of tumors, lymphatic metastasis is more common in ICC and is an important indicator for poor prognosis 22. Therefore, we further analyzed the expression of MUC1 in another 12 ICC samples with lymphatic metastasis (n = 6) or without lymphatic metastasis (n = 6) and found that the mRNA and protein levels of MUC1 were significantly higher in ICC cases with lymphatic metastasis compared to those without lymphatic metastasis (protein levels P value = 0.003; mRNA levels P value = 0.002; **Figure 1D**).

### High expression of MUC1 was associated with aggressive clinicopathological characteristics and poor prognosis after tumor resection

Tissue Microarray analysis (TMA) assay was further performed to detect MUC1 expression in ICC. Immunohistochemical data showed that MUC1 was rated as strong or moderate expression in 59.3% (127/214) of tumor tissues and 22.9% (49/214) in the corresponding adjacent normal intrahepatic bile duct tissue (**Figure 1C**). In order to explore the clinical significance of MUC1 in ICC, all 214 ICC patients were divided into MUC1 low (score negative or weak, n = 87) and MUC1 high (score moderate or strong, n = 127) groups based on immunohistochemical data. The high expression of MUC1 was significantly associated with the level of CA19-9 (P < 0.05), AFP (P < 0.05), and more aggressive tumor phenotypes including larger tumor size (P < 0.05), presence of lymph node metastases (P <0.05), vascular infiltration (P < 0.05), and advanced TNM stage (P < 0.05) (**Table 1**). High expression of MUC1 was also associated with OS and RFS after surgery (P= 0.002 for OS, P= 0.005 for RFS; **Figure 1E**). The median OS and RFS of patients in the MUC1 ^high^ group were significantly shorter than those in the MUC1 ^low^ group (OS, 16 vs. 29 months, P <0.01; RFS, 16 vs. 35 months, P <0.01). Multivariate analysis including all variables identified in Cox proportional hazard regression model further confirmed that the high expression of MUC1 was an independent prognostic factor for OS (hazard ratio [HR] = 1.59, 95% confidence interval [CI] 1.14-2.21, P = 0.006) and RFS (HR = 1.67, 95% CI 1.14-2.44, P = 0.009) (**Table 2**).

Furthermore, the predictive value of MUC1 was similar among patients with low recurrent risk, such as CA199 level <37 U/ml, tumor size ≤5 cm, without lymphatic metastasis, without vascular invasion and TNM stage I (**Figure S1**).

### Up-regulation of MUC1 promoted cell proliferation and invasion *in vitro*

**Figure 2:**
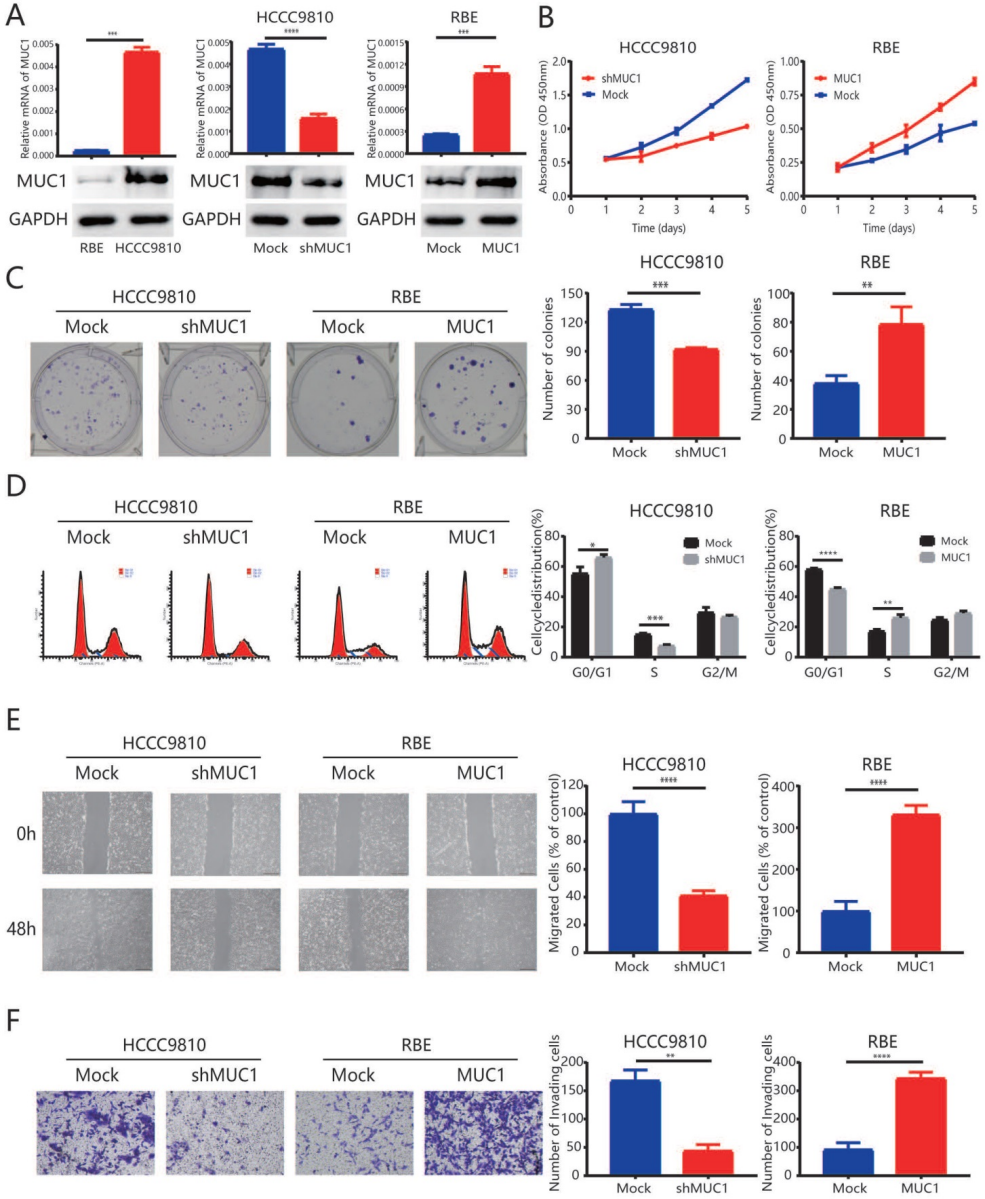
** Up-regulation of MUC1 promoted proliferation, cell cycle progression, and invasion of ICC *in vitro*. A.** The mRNA expression and protein expression of MUC1 in ICC cell lines (HCCC9810, RBE, HCCC9810-Mock, HCCC9810-shMUC1, RBE-Mock, and RBE-MUC1). **B.** Effects of MUC1 overexpression and down-regulation on proliferation using Cell Counting Kit-8 kit assay. **C.** Representative images of the clone formation assay. **D.** Representative images of MUC1 overexpression and down-regulation on cell cycle progression using flow cytometry after propidium iodide staining. **E.** Representative images of MUC1 overexpression and down-regulation on migration using scratch wound healing assay. Scale bars = 200 µm. **F.** Representative images of MUC1 overexpression and down-regulation on invasion using Matrigel invasion assay. Scale bars = 100 µm. All bar graphs depicted quantification of triplicate results with mean ± SD, *P < 0.05, **P < 0.01, ***P < 0.001.

To better understand the role of MUC1 in ICC pathogenesis, RBE cells were transfected with a lentiviral vector encoding wild-type MUC1 to over express the MUC1. On the other hand, HCCC9810 cells were transfected with a lentiviral vector encoding a short hairpin MUC1 (shMUC1) to decrease the MUC1 expression (**Figure 2A**). qRT-PCR and Western blot (**Figure 2A**) were used to confirm the efficiency of the transfection.

In the proliferation analysis, down-regulation of MUC1 in HCCC9810 cells resulted in a significant inhibition of tumor cell proliferation potential (P < 0.001, **Figure 2B and 2C**). In contrast, the proliferation of RBE-MUC1 cells was significantly enhanced compared to controls (P < 0.001, **Figure 2B and 2C**). Further cell cycle analysis showed that knocking down MUC1 expression in HCCC9810 cells significantly reduced the ratio of tumor cells in S phase. In contrast, a significant increase in cell cycle progression from G1 to S phase was observed in RBE cells overexpressed MUC1 (**Figure 2D**). In addition, both wound-healing analysis and Matrigel invasion analysis indicated that the mobility and invasion capacity significantly decreased in HCCC9810 cells after MUC1 knockdown while increased in RBE-MUC1 cells after MUC1 overexpression (**Figure 2E and 2F**).

### Up-regulation of MUC1 promoted ICC progression *in vivo*

**Figure 3:**
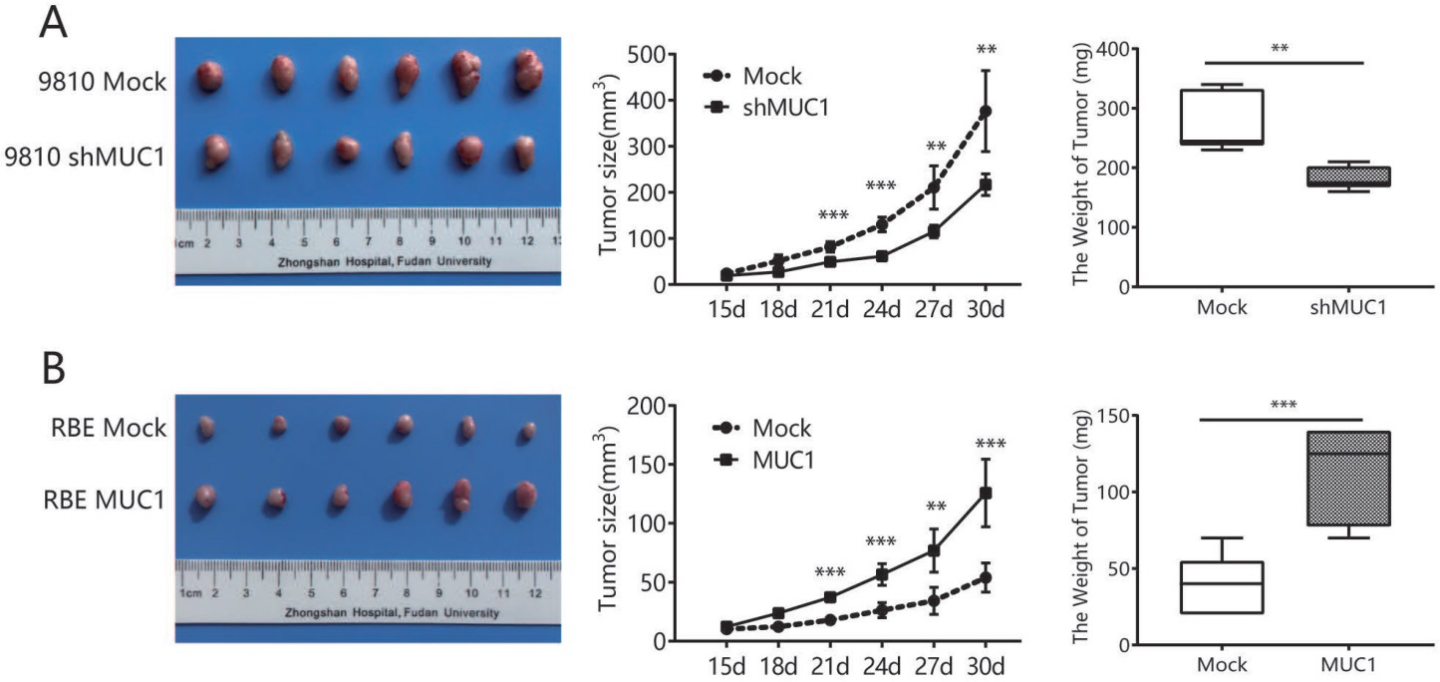
** Up-regulation of MUC1 promoted ICC progression *in vivo*. A and B.** Effects of MUC1 overexpression and down-regulation on the growth of *in vivo* subcutaneous xenograft tumors. Tumor volume and weight of xenografts derived from HCCC9810-shMUC1 cells were significantly reduced as compared with those of tumors derived from HCCC9810-Mock cells (n = 6); tumor volume and weight of xenografts derived from RBE-MUC1 cells were markedly increased as compared with those of tumors derived from RBE-Mock cells (n = 6). All bar graphs depicted quantification of triplicate results with mean ± SD. **P < 0.01, and ***P < 0.001.

Subsequently, a mouse subcutaneous xenograft model was developed to evaluate the effect of MUC1 on ICC progression *in vivo*. The tumor growth curve showed that tumors from HCCC9810-Mock and RBE-MUC1 cells grew significantly faster than tumors from HCCC9810-shMUC1 and RBE-Mock cells during the same period, respectively (**Figure 3A, 3B**). The tumor volumes of xenografts derived from HCCC9810-Mock and RBE-MUC1 cells were 376.3 ± 35.92 and 125.7 ± 11.75 mm^3^, respectively, which were significantly larger than those of HCCC9810-shMUC1 and RBE-Mock cells (217 ± 9.572 and 53.97 ± 5.058 mm^3^, all P value <0.01). Similarly, the weight of tumors derived from HCCC9810-Mock and RBE-MUC1 cells xenografts was significantly heavier than that of tumors derived from HCCC9810-shMUC1 and RBE-Mock cells, respectively (**Figure 3A and 3B**). Taken together, these results indicated that the expression of MUC1 could promote tumor gross.

### MUC1 regulated tumor cell proliferation, cell cycle progression, and invasive potential through the Wnt/β-catenin signaling pathway

**Figure 4:**
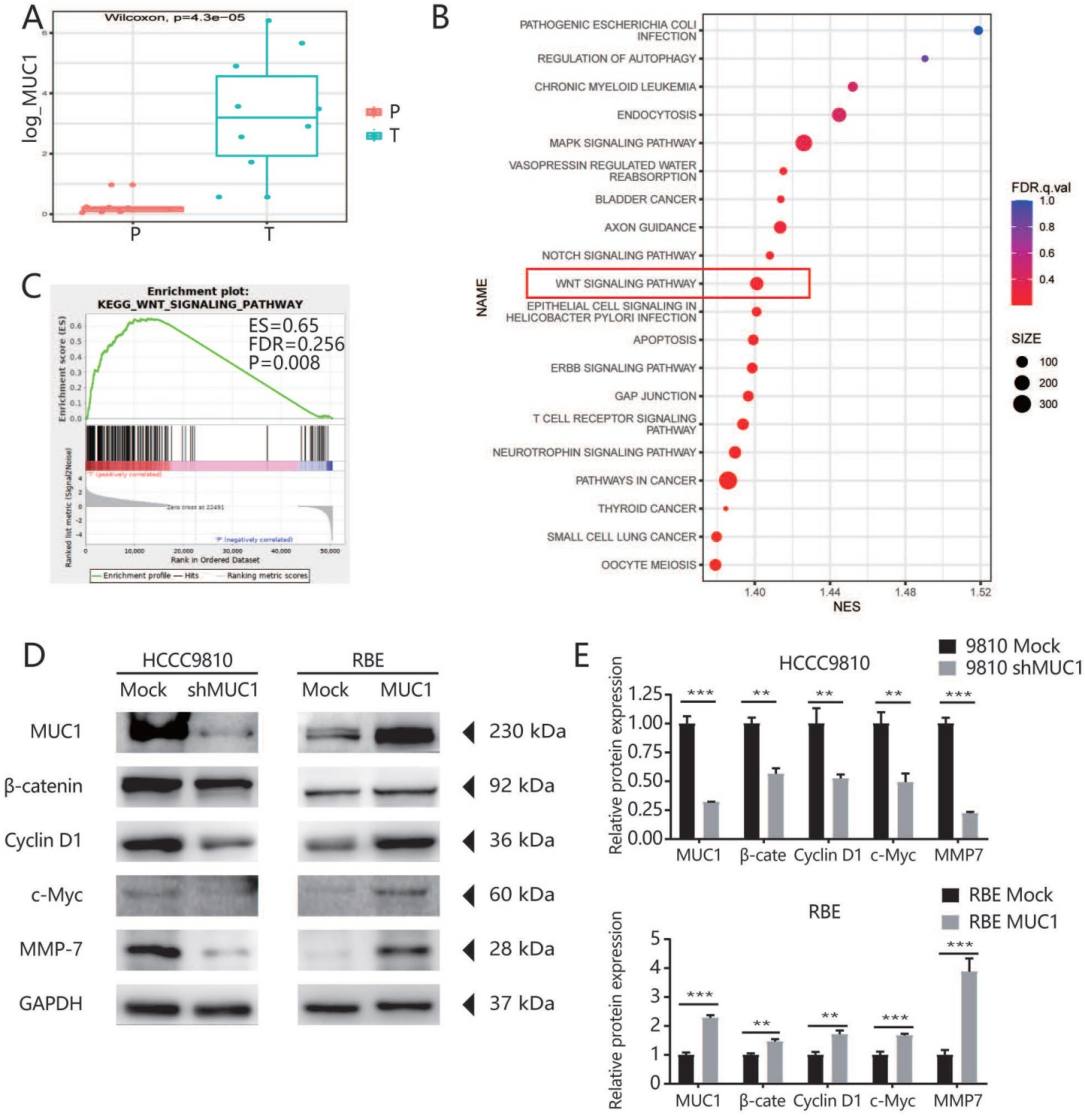
** MUC1 regulated ICC cell proliferation, cell cycle progression, and invasive potential through the Wnt/β-catenin signaling pathway. A.** High-throughput mRNA transcriptome sequencing was performed on 10 pairs of cancer tissues and adjacent tissues of ICC patients. In these sequencing samples, the mRNA expression level of MUC1 in the corresponding tumor tissues and adjacent tissues was subjected to paired T test. **B.** KEGG enrichment analysis based on these differentially expressed genes. **C.** GSEA analysis of WNT signaling pathway based on the gene expression profiles of high MUC1 group (red) versus low MUC1 group (blue) in our sequencing samples, ES, enrichment score; FDR, false discovery rate value. **D.** The expression level of related proteins in the Wnt/β-catenin signaling pathway, such as Cyclin D1, c-Myc, MMP-7, β-catenin and MUC1 were compared in indicated cells. GAPDH was used as loading control. **E.** Densitometry analysis was performed on three experiments representative of B and expressed relative to GAPDH or the corresponding total protein as the internal control.

**Figure 5:**
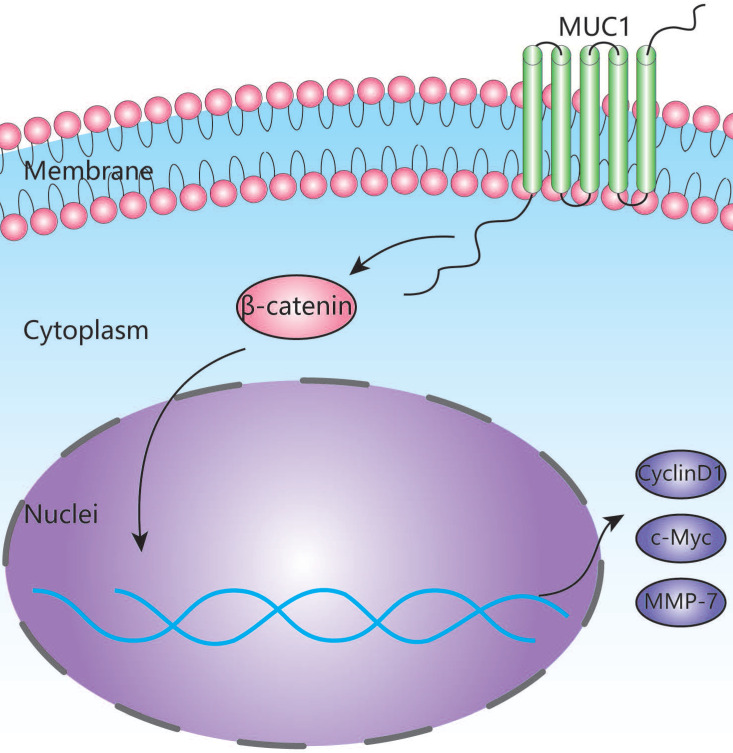
Schematic depiction of the mechanism underlying MUC1-mediated Wnt/β-catenin signaling pathway activation and ICC metastasis.

To further explore the potential mechanism of MUC1 in promoting tumor progression, we used RNA-seq assay to identify the transcription differences of 10 pairs of ICC patients' tumor tissues and adjacent tissues. As indicated by the qRT-PCR and West blot analysis, the mRNA expression level of MUC1 was significantly higher in tumor tissues compared with the adjacent tissues (**Figures 4A**). We further performed Gene Set Enrichment Analysis (GSEA) using Kyoto Encyclopedia of Genes and Genomes (KEGG) projects on the 10 pairs of ICC patients' tumor tissues and adjacent tissues, and found that the Wnt signaling pathway was significantly enriched (**Figure 4B**). Moreover, we found that the enrichment of Wnt signaling pathway was more prominent in high MUC1 group compared to low MUC1 group (FDR = 0.256, P = 0.008, **Figure 4C**). It has been known that β-catenin is an indispensable protein in the Wnt signaling pathway 23,24. Therefore, we further investigated the association between β-catenin and MUC1 expression in mRNA sequencing samples (**Figure S2**). Consistently, we observed significant differences in the expression of β-catenin mRNA between MUC1 Low group and MUC1 High group (0.64 ± 0.11 vs. 1.68 ± 0.33, respectively, P = 0.02). Further western blot analysis indicated that the expression level of β-catenin was significantly down-regulated in HCCC9810 after the inhibition of MUC1 expression, significantly up-regulated in RBE cells after overexpression of MUC1. Meanwhile, the expressions of key downstream target genes among the Wnt/β-catenin signal pathway such as Cyclin D1, c-Myc and MMP-7 25-27 were also explored. We found that the expression of Cyclin D1, c-Myc and MMP-7 was significant down-regulated in HCCC9810 after the inhibition of MUC1 expression, while these proteins were significantly up-regulated after overexpression of MUC1 in RBE cells (**Figure 4D, 4E**). In summary, these results suggested that MUC1 plays an important role in ICC progression by activating the Wnt/β-catenin signaling pathway (**Figure 5**).

## Discussion

ICC refers to a cholangiocarcinoma originating from peripheral bile ducts within the liver parenchyma and is distinguished from perihilar cholangiocarcinoma as well as distal cholangiocarcinoma 28. It accounts for approximately 5% to 10% of cholangiocarcinoma with dismal clinical outcome 29. The unsatisfying clinical outcomes might be a result of the high invasive feature of ICC including multifocal growth, regional lymph node metastasis and vascular invasions which all leading to poor long-term survival and short-term recurrence after resection 30.

Mucin 1 (MUC1) is a transmembrane glycoprotein that is aberrantly unregulated in numerous types of cancers, and serves as a key oncogene in the tumorigenesis of various human adenocarcinomas 31,32. However, the role of MUC1 in ICC progression as well as its potential mechanism remains poorly understood. Similarly as what had been proved in our previous study and other types of cancers 16,33-37, our data showed that the expression of MUC1 protein was significantly up-regulated in ICC tissues. More importantly, our results indicated that ICC patients with high MUC1 protein expression were associated with worse prognosis compared to those with low MUC1 protein expression. Notably, high protein expression of MUC1 was associated with more progressive clinical features such as larger tumor size, presence of lymphatic metastasis, advanced tumor stage, vascular invasion 28,38. In addition, the importance of MUC1 expression was similar among patients with low recurrent risk groups. Furthermore, our data also showed that knockdown of MUC1 expression could significantly suppressed tumor cell proliferation, invasion, migration, and cell cycle progression *in vitro*, and could significantly inhibit tumor growth in mouse subcutaneous xenograft model. Therefore, we believe that MUC1 plays an important role in tumor progression and might be a novel therapeutic target for ICC treatment.

It is well-known that the most ICC patients were associated with poor prognosis 28. It is very important to predict the risk of recurrence for timely postoperative adjuvant therapy to improve the prognosis of ICC patients 39. However, it is hard to predict which individual will have tumor relapse after surgical treatment for ICC patients 40. Our data showed that MUC1 was an independent predictor for overall survival and relapse-free survival after tumor resection. Furthermore, the prognostic significance of MUC1 was reconfirmed in the ICC subgroups with low recurrent risk (**Figure S1**), such as CA199 level <37 U/ml, Tumor size ≤5 cm, without lymphatic metastasis, without vascular invasion and TNM stage I 29,40. Thus, MUC1 might be a candidate biomarker for prognosis prediction of ICC patients.

To explore the potential mechanism of MUC1 in ICC, we further performed high-throughput mRNA transcriptome sequencing on surgical specimens of 10 ICC patients and found that the Wnt signaling pathway was significantly enriched in ICC tissues based on the KEGG enrichment analysis (**Figure 4B**). According to GSEA analysis 41, we also found that MUC1 plays an important role in the Wnt signaling pathway (**Figure 4C**). Interestingly, we found that the expression levels of β-catenin changed significantly with the expression level of MUC1 (**Figure S2**). Furthermore, the results from Western blot analysis also confirmed that the expressions of β-catenin as well as the key downstream genes (Cyclin D1, c-Myc, and MMP-7) in the Wnt/β-catenin signaling pathway were significantly affected by knockdown or overexpression of MUC1 in two ICC cell lines 25-27,42. Taken together, our data indicate that MUC1 might regulate Wnt/β-catenin signaling pathways to exert their tumor-activating functions in ICC (**Figure 5**). However, the detailed upstream mechanism of MUC1 regulation in ICC remains to be further explored.

This study has several limitations. First, this was a retrospective study from a single medical center which needs to be further validated in a large-scale, prospective, multicenter study. Meanwhile, although the association between MUC1 and Wnt/β-catenin signaling pathway was observed, how MUC1 triggers Wnt/β-catenin signaling activation is still unsolved and need to be further clarified.

In summary, our research shows that MUC1 is often up-regulated in ICC and promotes tumor progression by activating the Wnt/β-catenin signaling pathway. Importantly, the down-regulation of MUC1 significantly inhibited the proliferation and invasion of ICC. This study not only explored the pathogenesis, but also shed light upon identifying new therapeutic targets for ICC.

## Supplementary Material

Supplementary figures and table.

## Figures and Tables

**Table 1:** Correlation between MUC1 expression and clinicopathologic characteristics in 214 ICC patients

Clinicopathological indexes	MUC1		
	Low	High	P value*
**Age (year)**			
≤50	39	48	0.304
>50	48	79	
**Sex**			
Female	30	49	0.542
Male	57	78	
**HBsAg**			
Negative	39	77	**0.023**
Positive	48	50	
**AFP (ng/ml)**			
≤20	58	104	**0.01**
>20	29	23	
**CA19-9 (U/ml)**			
<37	51	48	**0.003**
≥37	36	79	
**ALT (U/l)**			
≤75	73	109	0.699
>75	14	18	
**Liver cirrhosis**			
No	73	96	0.142
Yes	14	31	
**Tumor size (cm)**			
≤5	47	39	**0.001**
>5	40	88	
**Tumor number**			
Single	78	115	0.829
Multiple	9	12	
**Tumor encapsulation**			
Complete	21	24	0.355
None	66	103	
**Lymphatic metastasis**			
NO	83	96	**0.000**
Yes	4	31	
**Vascular invasion**			
No	65	111	**0.017**
Yes	22	16	
**Tumor differentiation**			
I-II	54	92	0.109
III-IV	33	35	
**TNM stage**			
I	49	52	**0.027**
II-III	38	75	

AFP α-fetoprotein, CA19-9 carbohydrate antigen 19-9, ALT alanine aminotransferase; *χ^2^ tests for all analysis; P < 0.05, which indicates a significant difference.

**Table 2:** Univariate and multivariate analysis of prognostic factors in 214 ICC patients

Variables	OS		RFS	
	HR (95% CI)	P value	HR (95% CI)	P value
**Univariate analysis**				
Age (year) (>50 versus ≤50)	1.016 (1.002-1.030)	**0.023**	1.010 (0.994-1.027)	0.217
Sex (male versus female)	1.259 (0.917-1.728)	0.155	1.477 (1.005-2.169)	**0.047**
HBsAg (positive versus negative)	0.876 (0.670-1.146)	0.335	0.979 (0.704-1.360)	0.898
AFP (ng/ml) (>20 versus ≤20)	1.000 (1.000-1.000)	0.701	1.000 (1.000-1.000)	**0.007**
CA19-9 (U/ml) (≥37 versus <37)	1.000 (1.000-1.000)	**0.000**	1.000 (1.000-1.000)	**0.001**
ALT (U/L) (>75 versus ≤75)	1.001 (0.999-1.004)	0.261	1.002 (1.000-1.005)	0.108
Liver cirrhosis (yes versus no)	1.023 (0.653-1.602)	0.922	1.051 (0.620-1.780)	0.854
Tumor size (cm) (>5 versus ≤5)	1.069 (1.019-1.121)	**0.007**	1.095 (1.034-1.159)	**0.002**
Tumor number (multiple versus single)	1.072 (0.842-1.366)	0.572	0.957 (0.664-1.380)	0.815
Tumor encapsulation (none versus complete)	1.742 (1.178-2.576)	**0.005**	2.037 (1.262-3.288)	**0.004**
Lymphatic metastasis (yes versus no)	2.461 (1.729-3.503)	**0.000**	2.127 (1.367-3.308)	**0.001**
Vascular invasion (yes versus no)	1.151 (1.038-1.277)	**0.008**	1.124 (0.996-1.268)	0.059
Tumor differentiation (III-IV versus I-II)	1.016 (0.896-1.152)	0.808	1.018 (0.876-1.183)	0.819
TNM stage (II + III versus I)	1.441 (1.121-1.853)	**0.004**	1.037 (0.756-1.422)	0.823
MUC1 (low versus high)	1.652 (1.208-2.259)	**0.002**	1.655 (1.138-2.405)	0.008
**Multivariate analysis**				
Age (year) (>50 versus ≤50)	NA	NA	NA	NA
CA19-9 (U/ml) (≥37 versus <37)	**1.000 (1.000-1.000)**	**0.000**	**1.000 (1.000-1.000)**	**0.003**
Tumor size (cm) (>5 versus ≤5)	NA	NA	NA	NA
Tumor encapsulation (none versus complete)	NA	NA	**1.899 (1.162-3.106)**	**0.011**
Lymphatic metastasis (yes versus no)	**2.105 (1.426-3.107)**	**0.000**	NA	NA
Vascular invasion (yes versus no)	NA	NA	**1.726 (1.095-2.719)**	**0.019**
TNM stage (II + III versus I)	NA	NA	NA	NA
MUC1 (low versus high)	**1.587 (1.140-2.209)**	**0.006**	**1.665 (1.135-2.443)**	**0.009**

Cox proportional hazards regression model. Variables for multivariate analysis were adopted for their prognostic significance by univariate analysis (P<0.05), and these variables were assessed for prognostic significance by univariate analysis with forward stepwise selection (forward, likelihood ratio); HR hazard ratio, 95% CI 95% confidence interval, AFP α-fetoprotein, CA19-9 carbohydrate antigen 19-9, ALT alanine aminotransferase, NA not applicable; P values are all < 0.05, which indicate significantly difference.

## Abbreviations

ICC: intrahepatic cholangiocarcinoma
MUC1: Mucin1
CCK8: Cell Counting Kit-8
TMA: tissue microarrays analysis
TNM: tumor-node-metastasis
FCM: flow cytometry
FBS: fetal bovine serum
ES: enrichment score
FDR: false discovery rate value
NES: Normalized Enrichment Score
β-catenin: catenin beta interacting protein 1
c-Myc: MYC proto-oncogene
KEGG: Kyoto Encyclopedia of Genes and Genomes
MMP-7: matrix metallopeptidase 7
FPKM: Fragments Per Kilobase of exon model per Million mapped fragments
GAPDH: glyceraldehyde 3-phosphate dehydrogenase
PI: propidium iodide
