# Supplementary material for: Mucin 1 promotes tumor progression through activating WNT/β-catenin signaling pathway in intrahepatic cholangiocarcinoma
Source: J Cancer. 2021 Oct 3;12(23):6937–47. doi: 10.7150/jca.63235 (PMC8558653; doi:10.7150/jca.63235)
Supplement: Supplementary file 1 — Supplementary figures and table. [file jcav12p6937s1.pdf]

Supplementary Figure S1.

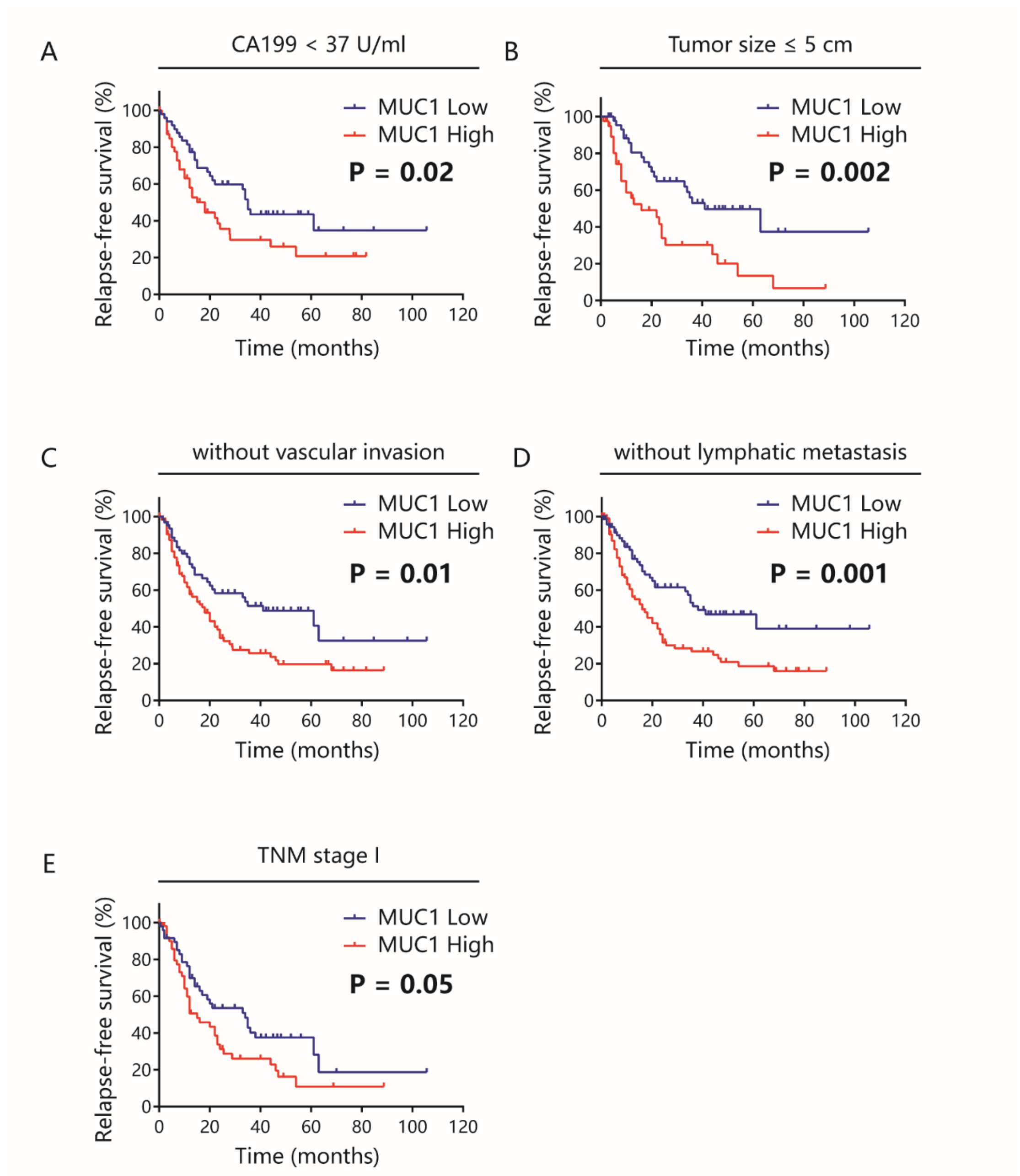

Prognostic significance assessed using Kaplan–Meier survival estimates and log-rank tests. Comparison of relapse-free survival between the MUC1 low and MUC1 high group in different subgroups.

Supplementary Figure S2.

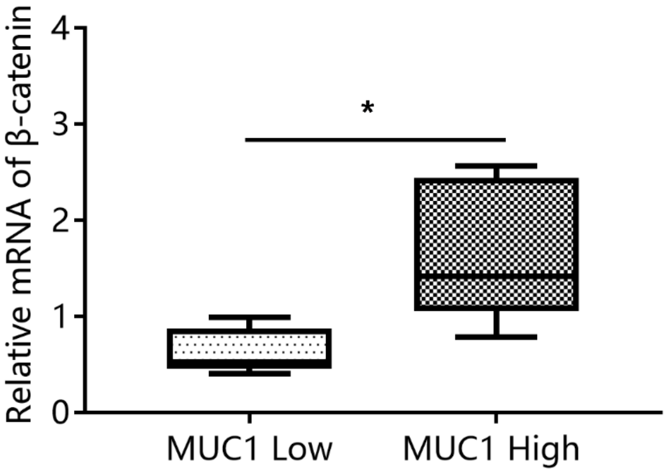

According to the mRNA expression of MUC1 in tumor tissues sequenced from 10 patients, they were divided into MUC1 Low group (n=5) and MUC1 High group (n=5). The mRNA expression is presented in the form of FPKM of RNA-seq sequencing results. There were significant differences in the expression of β-catenin mRNA between the two groups (0.64 ± 0.11 vs. 1.68 ± 0.33, respectively, * P = 0.02).

Supplementary Table S1

10 pairs of ICC patient clinical information of sequenced specimens

Patient code	Gender	Age	Date of surgery	Tumor type	Tumor diameter (cm)	Liver capsule invasion	Lymph node metastasis	Microvascular infiltration
T009	M	44	2018/12/13	ICC	9.5	Yes	No	No
T015	M	61	2019/1/3	ICC	8	Yes	No	No
T017	F	70	2019/1/9	ICC	10.5	No	No	No
T021	M	71	2019/1/30	ICC	11	No	No	Yes
T024	F	54	2019/2/22	ICC	7	No	Yes	No
T027	M	51	2019/2/27	ICC	8	Yes	No	No
T033	M	72	2019/5/8	ICC	5	No	No	No
T036	F	68	2019/5/8	ICC	4	No	Yes	Yes
T039	F	72	2019/5/10	ICC	3.5	No	Yes	No
T042	M	63	2019/5/10	ICC	11.5	No	No	Yes
